# Telomere Length and Hearing Loss: A Two-Sample Mendelian Randomization

**DOI:** 10.3390/ijerph19158937

**Published:** 2022-07-22

**Authors:** Yun Liu, Shuangyan Liu, Jiarui Xin, Peiyi Qian, Shuli Guo, Xiaojun Xu, Dahui Wang, Lei Yang

**Affiliations:** 1 School of Public Health, Hangzhou Normal University, Hangzhou 310000, China; 2020111012037@stu.hznu.edu.cn (Y.L.); xinjiarui@stu.hznu.edu.cn (J.X.); 2020112012100@stu.hznu.edu.cn (P.Q.); 2020011012014@stu.hznu.edu.cn (S.G.); 2021111012046@stu.hznu.edu.cn (X.X.); 2 School of Public Health, Tongji Medical College, Huazhong University of Science and Technology, Wuhan 430030, China; d202181610@hust.edu.cn

**Keywords:** Mendelian randomization, telomere length, hearing loss, causal effect, age-related hearing loss, noise-induced hearing loss

## Abstract

Background: Observational studies have suggested that there may be an association between telomere length (TL) and hearing loss (HL). However, inferring causality from observational studies is subject to residual confounding effects, reverse causation, and bias. This study adopted a two-sample Mendelian randomization (MR) approach to evaluate the causal relationship between TL and increased risk of HL. Methods: A total of 16 single nucleotide polymorphisms (SNPs) associated with TL were identified from a genome-wide association study (GWAS) meta-analysis of 78,592 European participants and applied to our modeling as instrumental variables. Summary-level data for hearing loss (HL), age-related hearing loss (ARHL), and noise-induced hearing loss (NIHL) were obtained from the recent largest available GWAS and five MR analyses were used to investigate the potential causal association of genetically predicted TL with increased risk for HL, including the inverse-variance-weighted (IVW), weighted median, MR-Egger regression, simple mode, and weighted mode. In addition, sensitivity analysis, pleiotropy, and heterogeneity tests were also used to evaluate the robustness of our findings. Results: There was no causal association between genetically predicted TL and HL or its subtypes (by the IVW method, HL: odds ratio (OR) = 1.216, *p* = 0.382; ARHL: OR = 0.934, *p* = 0.928; NIHL: OR = 1.003, *p* = 0.776). Although heterogenous sites rs2736176, rs3219104, rs8105767, and rs2302588 were excluded for NIHL, the second MR analysis was consistent with the first analysis (OR = 1.003, *p* = 0.572). Conclusion: There was no clear causal relationship between shorter TLs and increased risk of HL or its subtypes in this dataset.

## 1. Introduction

Hearing loss (HL) is defined as the deterioration of hearing acuity, usually manifested as an increase in the hearing threshold. The exact cause of HL is not yet known. It is currently believed to be caused by a combination of genetics, environmental factors, lifestyle, and others. Common risk factors include congenital disease, age, noise exposure, use of ototoxic drugs and solvents, chronic inflammation, oxidative stress injuries, diabetes, and hypertension [1,2,3,4]. Age-related hearing loss (ARHL) and noise-induced hearing loss (NIHL) are the most common subtypes, and HL has a variety of adverse effects on health and is significantly increased with advancing age, with HL also being described as an independent risk factor for dementia [5]. According to the World Health Organization (WHO) estimates from 2019, about 1.57 billion people worldwide suffer from hearing loss with this number likely to increase to more than 2.45 billion in 2050 [6]. Global burden of disease research has shown that HL is the third leading cause of disability in the world [7], and when we evaluate the cost associated with the number of years associated with HL-mediated disability, the most recent data suggest that it may exceed USD 750 billion per annum [8]. This means that as the absolute number and percentage of the aging population grow, HL will become a major cause of disability.

Telomeres are nucleoprotein complexes located at the ends of chromosomes designed to protect their durability and integrity [9]. With each cellular division, telomeres shorten [10], and this shortening can lead to gene mutations, leading to DNA damage, increased apoptosis, and senescence [10]. Telomere length (TL) has also been associated with several age-related diseases and is considered a marker of biological aging [9,11] and a potential indicator for chronic disease [12]. Telomeres can be extracted from peripheral blood leukocytes, and their heritability has been reported to range from 36% to about 84% [13]. Observational studies have also found that TL may be related to HL [14,15,16,17,18], with one set of gene sequencing data of familial HL revealing that mutation in the telomere-related gene *DFNA25* was a risk factor for HL [16]. In addition, a case-control study showed that individuals in the highest quartile for relative TL had a 47% lower risk of NIHL than individuals in the lowest quartile [15]. However, other studies have not widely replicated this result, and a cross-sectional survey did not find any association between TL and HL [19]. These contradictory data are likely a byproduct of the fact that evidence from human observational studies is susceptible to reverse causality and confounding factors, making it difficult to resolve whether there is a causal relationship between TL and HL.

Given these issues, we decided to use a Mendelian randomization (MR) approach to evaluate the potential causal association between TL and the risk of HL and its subtypes. MR is an epidemiological method [20,21], which uses the advantages of germline DNA variation (stability and random assortment of alleles) to generate so-called “instrumental variables (IV)” as a proxy for exposure. This concept is similar to the random design and minimizes the bias caused by confounding factors and reverse causality. In addition, MR is more cost effective than randomized controlled trials and is also more convenient, time-effective, and not subject to ethical restrictions. This method is more likely to resolve accurate causal relationships [22]. Thus, we used MR to explore the causal relationship between TL and HL and its subtypes. These data might help clarify the underlying factors behind the etiology of HL and aid in the development of future prevention and intervention strategies.

## 2. Materials and Methods

### 2.1. Genetic Instrument Selection

We obtained the data from 78,592 previous GWAS participants from the European Network for Genetic and Genomic Epidemiology (ENGAGE), the European Prospective Investigation into Cancer and Nutrition (EPIC) Cardiovascular Disease (CVD) and InterAct studies, to extract data for 52 previously identified common SNPs associated with TL as identified by the GWAS meta-analysis of leukocyte TL (including allele frequency, *β* value, *SE*, and *p*-value, Supplementary Table S1). We were then left with 21 variants of interest following GWAS significance adjustment (*p* < 5 × 10^−8^). We subsequently used linkage disequilibrium (LD) to determine whether any of these SNPs were genetically linked and set our exclusion values at an r^2^ value of 0.001 [23] and a window size of 10,000 kb. The LD proxies were defined using 1000 genomes of European ancestry, and SNPs were excluded if not present in the outcome GWAS. At the same time, we evaluated each instrument SNP and their proxies in the PhenoScanner GWAS database (http://www.phenoscanner.medschl.cam.ac.uk/ (accessed on 12 July 2022)) to assess their associations (*p* < 5 × 10^−8^) with potential confounding traits among HL and TL and manually removed these SNPs from the MR analysis to satisfy the second assumption (no confounders existing). A flow chart of the step-by-step MR analytical process is shown in Supplementary Figure S4. We then assessed whether there was any weak instrumental variable bias, that is, we determined if any of the selected genetic variations were weakly correlated with exposure, applying the F statistic [24]: $(F=R^{2}\;\left(n-k-1\right)/k\left(1-R^{2}\;\right)$, where *R*^2^ is the variance of exposure explained by selected instrumental variables, n is the sample size, and k is the number of instrumental variables. F statistic exceeding 10 indicates potential to explain the phenotypes [20]. Statistical power was calculated using “mRnd”, a publicly available online tool [25].

### 2.2. Genetic Summary Data for Hearing Loss

We obtained our causal estimates between exposure and outcomes using the two-sample MR method. We unified the exposure and outcome SNPs from the same allele and dropped all palindromic SNPs from the analysis. We used publicly available GWAS summary statistics from OpenGWAS (https://gwas.mrcieu.ac.uk/ (accessed on 12 July 2022)). The GWAS summary data for HL from FinnGen biobank included 94,570 participants of European ancestry (6730 cases and 87,840 controls), while the ARHL GWAS summary data from FinnGen biobank included 88,448 participants of European descent (608 cases and 87,840 controls), and the NIHL GWAS summary data from UK Biobank included 453,482 participants of European ancestry (171,586 cases and 281,896 controls. Participants were assigned case/control status based on whether the participant reported hearing difficulty or problems with background noise). The relevant institutional review boards approved all studies, and all participants provided written informed consent.

### 2.3. Mendelian Randomization Estimates

**Figure 1:**
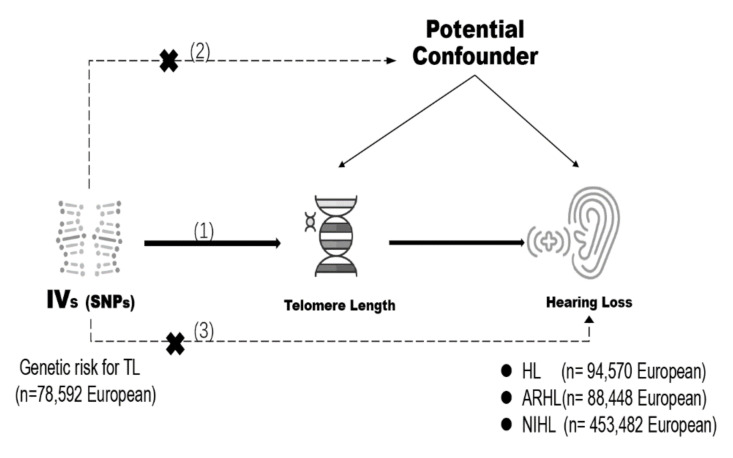
Schematic diagram of the two-sample Mendelian randomization analysis regarding the association of genetically predicted telomere length with risk of hearing loss and its subtypes.

MR uses instrumental variables to assess the causal relationship between a given exposure and a specific outcome [20,26,27]. MR can prevent confounding and reverse causation biases in conventional observational studies, and it relies on three assumptions [28,29]: (1) The instrumental variables need to be significantly associated with TL, (2) the instrumental variables must not be associated with any other factors associated with both TL and HL risk, and (3) the instrumental variables must only affect HL risk via TL (Figure 1).

### 2.4. Statistical Analysis

We only included genetic variants in these analyses that were available in all exposure SNPs or their proxies and in the outcome dataset and after removing the palindromic SNPs from these datasets. We addressed the first assumption (a true association between SNPs and TL) by selecting SNPs that strongly predicted TL. We then excluded any traits without suitable genetic instruments leaving a total of 6 SNPs which were included in the MR analyses with HL and ARHL, 15 SNPs in the MR analyses with NIHL. Five MR methods were then used to assess the relationships between TL and HL subtypes, including the inverse-variance-weighted (IVW), weighted median (WM), simple median, weighted simple median, and Mendelian randomization-Egger (MR-Egger) methods [20]. We also completed a series of sensitivity analyses using conventional IVW, WM, MR-egger, and MR Pleiotropy RESidual Sum and Outlier (MR-PRESSO) to evaluate the robustness of our findings.

This overlapping approach allowed us to leverage the advantages of each MR method, so they could complement each other and provide more reliable causal effect data. IVW is the earliest and most commonly used method for two-sample MR analysis. The MR-Egger regression test intercept evaluates the evidence for directional pleiotropy, i.e., the “Instrument Strength Independent of Direct Effect (InSIDE)” assumption, where intercepts that are significantly different from zero suggest directional pleiotropy [30]. WM allows some variants to be invalid instruments provided at least half are valid instruments [31] and we used MR-PRESSO to detect and correct for horizontal pleiotropic outliers [32]. In addition, if significant heterogeneity existed in the research, then the random-effects model was used. Our statistical analyses were performed using the “TwoSampleMR” and “MR-PRESSO” packages in R v3.6.3 software [32,33], and significance was set at *p* < 0.0167 (*p* = 0.05/3 outcomes = 0.0167).

## 3. Results

### 3.1. Selection of Instrumental Variables

Previously reported meta-analysis of GWAS data from participants of European ancestry identified 52 potential SNPs for this evaluation. Our data validation reduced this to 16 SNPs related to TL, which were also independent of any potential confounding factors and then applied as instrumental variables in our MR evaluations to identify any causal relationships between TL and HL [29,34,35]. The proportion of variance in average TL explained by individual SNPs ranged from 0.08% to 0.36% [35], and the F statistic of these SNPs was much greater than 10, indicating that the possibility of weak instrumental variable deviation was low. The characteristics of these SNPs are summarized in Table 1.

### 3.2. MR Analysis of TL with Risk of HL and Its Subtypes

**Figure 2:**
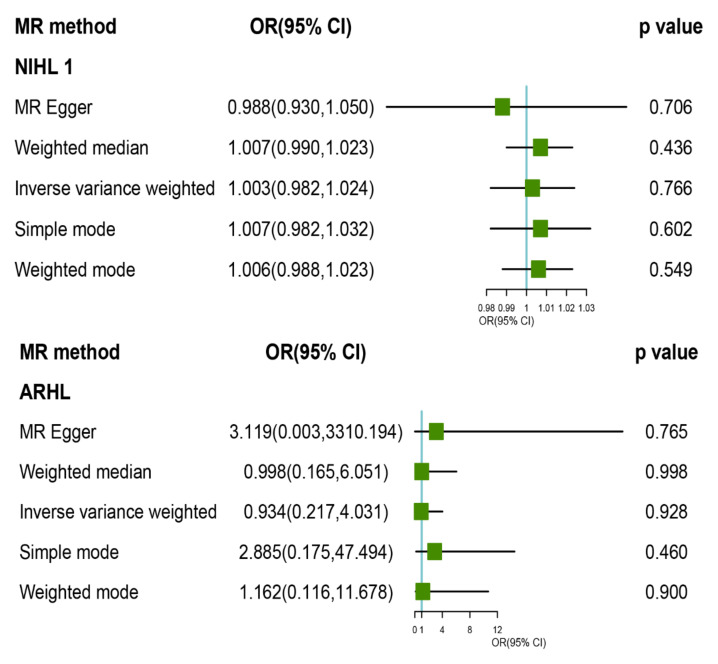

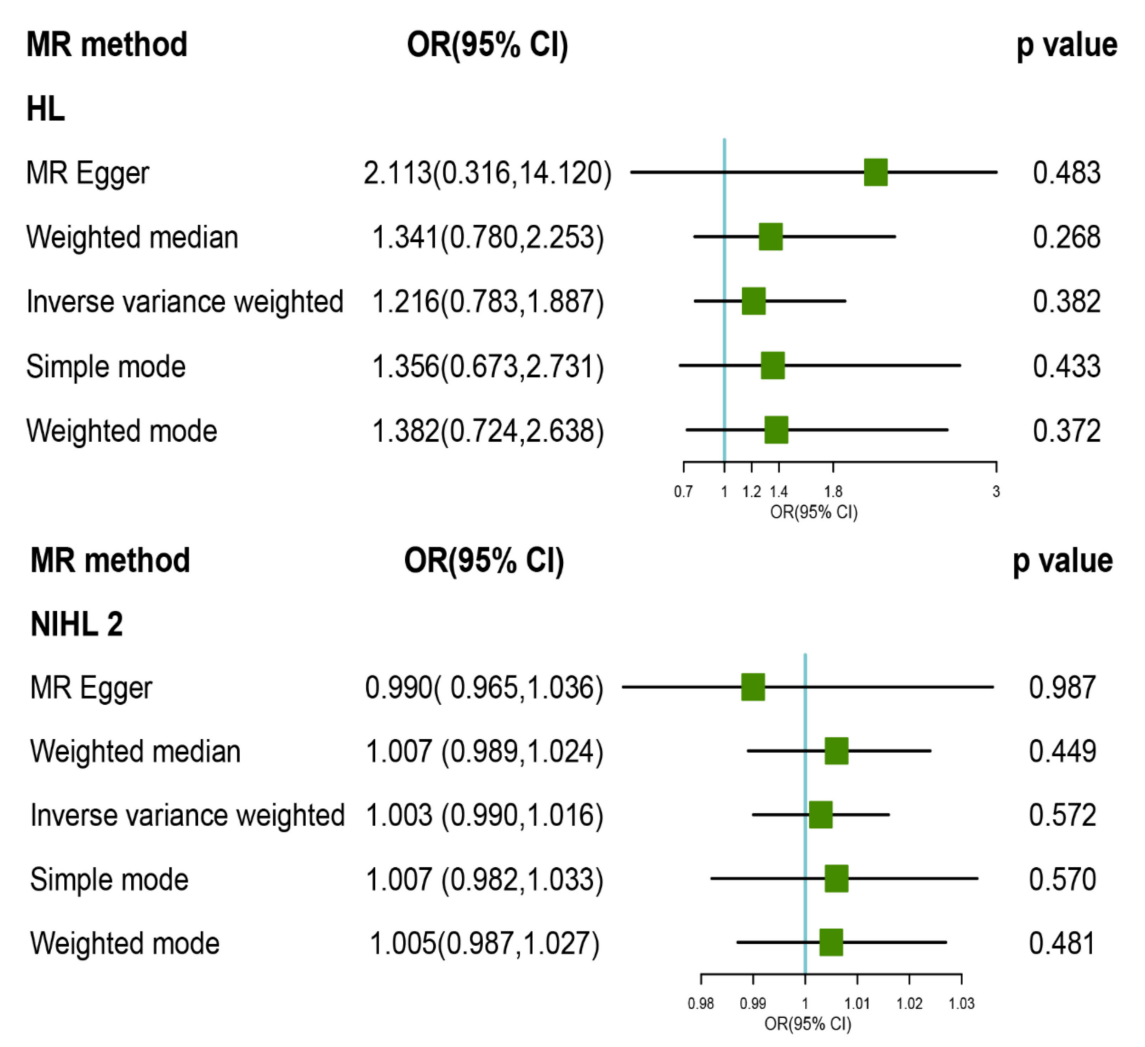
Mendelian randomization (MR) results for telomere length and hearing loss (HL) and its subtypes. OR: odds ratio; CI: confidence interval; HL: all hearing loss; ARHL: age-related hearing loss; NIHL: noise-induced hearing loss. A, HL; B, ARHL; C, NIHL; D, NIHL excluding the following four heterogeneous sites: rs2736176, rs3219104, rs8105767, and rs2302588 post-MR.

We selected several independent SNPs related to TL from the genetic data associated with participants of European ancestry to perform a two-sample MR analysis to evaluate their causal relationship with HL and its subtypes and removed any sites with heterogeneity and non-palindromic SNPs. Our two-sample MR analysis used the IVW, WM, MR-Egger regression, simple mode, and weighted mode to evaluate the interactions between these inputs and outcomes, and none of these evaluations identified any causal relationship between genetically predicted TL and risk for HL, ARHL, or NIHL (by the IVW method, HL: odds ratio (OR) = 1.216, *p* = 0.382; ARHL: OR = 0.934, *p* = 0.928; NIHL: OR = 1.003, *p* = 0.776). The MR estimates of TL in HL using conventional MR analysis are presented in Figure 2.

### 3.3. Pleiotropy and Sensitivity Analysis

We then used IVW, WM, MR-Egger, and MR-PRESSO methods to calculate causal estimates of TL on HL risk using heterogeneity and pleiotropy and then confirmed these findings using a sensitivity analysis of the causal associations between TL and HL and its subtypes. MR-PRESSO tests suggested potential directional pleiotropy in the causal relationship between TL and NIHL (*p* < 0.001) while the Cochran *Q* test and *I*^2^ statistics suggested potential heterogeneity and pleiotropic effects in these values (IVW: *Q* = 41.55, *df* = 14, *I*^2^ = 65.6%, *p* = 0.0001; MR-Egger: *Q* = 40.69, *df* = 13, *I*^2^ = 66.3%, *p* = 0.001; Supplementary Figure S2). We then excluded four heterogeneous sites (rs2736176, rs3219104, rs8105767, and rs2302588) in a second round of MR and found that this heterogeneity disappeared in the second round of MR for NIHL (IVW: *p* = 0.73; MR-Egger: *p* = 0.65) and pleiotropic disappearance (MR-PRESSO: *p* = 0.779; Table 2, Supplementary Figure S3). We also conducted a combined analysis for the SNPs associated with NIHL using a fixed effects model (*p* = 0.765, OR = 1.003, 95% CI: 0.982–1.024) and simultaneously used the “leave one out method” to confirm that our observations were stable (Supplementary Figure S4). These analyses verified the stability of our findings (Table 2).

## 4. Discussion

We conducted two-sample MR studies to determine whether TL phenotypes (exposures) were potentially causally related to HL subtypes (the outcome). Our results suggested that there was no cause–effect relationship between TL and the risk of HL, and that, in the absence of heterogeneity and unknown pleiotropy effects, this method provides robust causal estimates. Nevertheless, multiple lines of evidence suggest that TL is robustly associated with HL risk [14,15]. In a case-control study, categorical analyses revealed that subjects within the highest TL tertiles were at a lower risk for ARHL when compared to those in the lowest and middle tertiles (OR = 0.327, 95% CI: 0.170–0.629, *p* = 0.0008) [14]. There was also a descending trend of TL as the degree of pure tone threshold average was reduced suggesting that ARHL might be associated with telomere attrition. A recent study reported that individuals in the top quartile of TL have a 47% lower hearing loss risk than those in the bottom quartile (OR = 0.53, 95% CI: 0.38–0.74), with this decline in risk growing to 55% (OR = 0.45, 95% CI: 0.28–0.73) in females [15]. These results suggest that TL is closely associated with HL in general, particularly in females with mild HL. However, because it can be challenging to obtain individual-level data from multiple cohorts, we could not complete a sex-stratified MR analysis in this study. In addition, cross-sectional studies have been unable to demonstrate a relationship between TL and HL, suggesting that TL is not associated with HL (children: OR = 0.99, 95% CI: 0.55–1.78; adult: OR = 1.35, 95% CI: 0.81–2.25) [19], which is consistent with our findings. Despite conflicting evidence and a lack of longitudinal studies supporting the relationship between TL and HL, many researchers have highlighted the potential association between these events but have not evaluated the cause–effect relationships between these observations. To the best of our knowledge, our study is the first to use the MR method to evaluate these relationships, and we found no significant correlation between genetic markers for TL and HL and its subtypes, suggesting that TL may not be directly associated with HL.

Our findings could be explained by several potential mechanisms. HL can be multifactorial, caused by genetic, environmental, medication, and lifestyle factors [36,37], and the pathogenesis of HL, ARHL, and NIHL always involves free radical production, ion imbalance, excitotoxicity, oxidative stress, and inflammatory response [3,4,38]. Several studies have shown that ROS can directly damage DNA irrespective of TL thus inducing the DNA damage response and senescence [39,40]. Chronic inflammation and ROS have also been shown to cause cell dysfunction without any obvious shortening of the telomeres [41], and while recent reports have shown that telomere shortening is likely to be a critical factor in the genetic profile of cells as they age, these are not necessarily causal effects. A recent report indicated that telomere shortening is not believed to be directly involved in other signs of aging but only a regulator of the genetic changes in human gene expression associated with cellular aging, with the majority of the genes affected by TL involved in apoptosis and cell death [12]. Similarly, there have been several observational studies that have suggested a potential link between TL and various cancers and non-neoplastic diseases, but none of these reported any causality when evaluated by MR, with these outcomes likely the result of the sensitivity of observational studies to confounding effects and reverse causality [29,34,42,43]. At present, there is insufficient evidence to suggest that TL is the driving etiological factor in many diseases, and the pathogenic link between TL and HL needs to be evaluated in greater detail. Follow-up studies should also include any new SNPs related to genetic variation associated with telomeres and HL as it is necessary to keep the relevant instrumental variables up to date in an effort to produce more reliable conclusions.

To the best of our knowledge, our study was the first to apply MR analysis to HL, and there are several important strengths. First, compared with traditional observational research, the causal association of TL and HL was not distorted by reverse causal associations and confounding factors since the genetic variations related to TL are randomly distributed among the population at birth. Second, the F statistic implying the possibility of weak instrument variable bias was low. Third, our results were robust when the MR evaluation completed after removing the heterogeneous sites also reported no demonstrable causal relationship between TL and HL. Subsequent application of the “leave one out method” also confirmed the generally robust nature of our results and highlighted the application of this approach to causal relationship evaluations for genetic traits. In addition, the MR-Egger regression results show that there is no horizontal pleiotropy among the instrumental variables, indicating that our conclusions are both robust and credible.

However, there are some limitations to this study. First, the use of the PhenoScanner database found that rs2736176 is associated with rheumatoid arthritis, which is involved in HL [44,45], although a causal relationship was still not found between TL and HL after removing this SNP. Second, there may be unidentified pleiotropic effects in these datasets, so future investigators should analyze more MR methods and collect more data to adjust for confounding risk factors, such as LDSC (Linkage disequilibrium score) regression [46], CAUSE (Causal Analysis Using Summary Effect Estimates) [47], and GRAPPLE (Genome-wide mR Analysis under Pervasive PLEiotropy) [48]. Third, we measured leukocyte TL; however, several studies have shown a strong correlation between the TL in different tissues and pathogenic outcomes [12,49], and we did not obtain full summary GWAS information of telomere which renders it difficult to explore a possible bidirectional association between telomeres and hearing loss (hearing loss may be causally associated with telomere length). In addition, many of the diagnostic criteria for hearing loss in GWAS are based on self-reported hearing status rather than audiometry, which may lead to bias. Last, although the whole study population was of European decent, European populations are ethnically heterogeneous, and so our study population may not be fully representative.

## 5. Conclusions

This study is the first to use MR to evaluate the causal relationship between TL and HL and its subtypes, with the view to determining the causal relationship between TL and HL using a two-sample MR analysis. Our data did not identify any causal relationship between TL and NIHL, which suggests that the prevention and control measures used to combat changes in TL may not benefit HL, and that it cannot be used as an indicator for monitoring early HL. However, given the continued development of large-scale, multi-center GWAS cohort studies, in follow-up studies, novel genetic variant-related SNPs associated with telomeres and HL should be reevaluated using this model to ensure that our evaluations use the most up to date instrumental variables to complete MR analysis. This study also reveals that this is a valuable tool for identifying causal relationships and may help to identify other causal relationships in the future.

## Figures and Tables

**Table 1:** Characteristics of SNPs predictive of telomere length (TL).

SNP	Chr	Nearby Gene	*EA*	*MAF*	*β*	*SE*	*p*-Value	*F* Statistic
rs10936600	3	*LRRC34*	T	0.243	−0.086	0.006	6.00 × 10^−51^	225.300
rs7705526	5	*TERT*	A	0.328	0.082	0.006	5.00 × 10^−45^	198.300
rs4691895	4	*NAF1*	C	0.783	0.058	0.006	1.00 × 10^−21^	91.000
rs9419958	10	*STN1*	C	0.862	−0.064	0.007	5.00 × 10^−19^	79.500
rs75691080	20	*STM3*	T	0.091	−0.067	0.009	6.00 × 10^−14^	56.500
rs59294613	7	*POT1*	A	0.293	−0.041	0.005	1.00 × 10^−13^	55.100
rs8105767	19	*ZNF257*	G	0.289	0.039	0.005	5.00 × 10^−13^	52.100
rs3219104	1	*PARP1*	C	0.830	0.042	0.006	9.00 × 10^−11^	42.000
rs2736176	6	*AIF1*	C	0.313	0.034	0.005	3.00 × 10^−10^	39.400
rs3785074	16	*TERF2*	G	0.263	0.035	0.006	4.00 × 10^−10^	38.900
rs7194734	16	*MPHOSPH6*	T	0.782	−0.037	0.006	7.00 × 10^−10^	38.100
rs228595	11	*ATM*	A	0.417	−0.028	0.005	1.00 × 10^−8^	32.200
rs2302588	14	*DCAF4*	C	0.100	0.048	0.008	2.00 × 10−^8^	31.900
rs13137667	4	*MOB1B*	C	0.959	0.077	0.014	2.00 × 10^−8^	31.200
rs55749605	3	*SENP7*	A	0.579	−0.037	0.007	2.00 × 10^−8^	31.200
rs62053580	16	*RFWD3*	G	0.169	−0.039	0.007	4.00 × 10^−8^	30.200

Note: SNP: single-nucleotide polymorphism; Chr: chromosome; *EA*: effect allele; *MAF*: minor allele frequency; *SE*: standard error; *β*: standard deviation change in leukocyte TL per copy of the effected allele.

**Table 2:** Mendelian randomization estimates for the association between TL and HL and its subtypes.

HLs	IVW					WM		MR Egger							MR_ PRESSO
	OR (95% CI)	*p*	Cochran *Q* Statistics (*df)*	*I* ^2^	*p*	OR (95% CI)	*p*	OR (95% CI)	*p*	Intercept (se)	*p*	Cochran *Q* Statistics (*df)*	*I* ^2^	*p*	*p*
HL	1.216 (0.783, 1.887)	0.382	2.449 (5)	−1.042	0.784	1.341 (0.780, 2.253)	0.268	2.113 (0.316, 14.120)	0.483	−0.027 (0.046)	0.589	2.106 (4)	−1.849	0.716	0.866
ARHL	0.934 (0.217, 4.031)	0.928	5.18 (5)	0.035	0.393	0.998 (0.151, 6.593)	0.998	3.119 (0.003, 3310.194)	0.765	−0.058 (0.167)	0.745	5.037 (4)	−0.191	0.284	0.520
NIHL	1.003 (0.982, 1.024)	0.766	41.549 (14)	0.639	0.000	1.007 (0.99, 1.023)	0.436	0.988 (0.93, 1.05)	0.706	0.001 (0.002)	0.609	40.687 (13)	0.631	0.0001	<0.001
NIHL^a^	1.003 (0.990, 1.016)	0.572	7.844 (11)	−0.402	0.727	1.007 (0.989, 1.024)	0.449	0.990 (0.965, 1.036)	0.987	0.000 (0.001)	0.818	7.788 (10)	−0.412	0.65	0.779

Note: IVW: inverse variance weighting method; WM: weighted median method; MR-Egger: Mendelian randomization Egger method; OR: odds ratio; CI: confidence interval, NIHL^a^ refers to outcomes following the elimination of the four heterogenous positions at rs2736176, rs3219104, rs8105767, rs2302588 based on the results of the adjusted MR.

## Data Availability

The data used in this study are publicly available and can be accessed via the references in the manuscript.

## Supplementary Materials

The following supporting information can be downloaded at: https://www.mdpi.com/article/10.3390/ijerph19158937/s1, Supplementary Figure S1. Forest plots describing the Mendelian randomization analyses evaluating the association between telomere length (TL) and various hearing loss (HL) types. A, HL; B, age-related HL; C, noise-induced HL (NIHL); D, NIHL excluding heterogeneous sites rs2736176, rs3219104, rs8105767, and rs2302588. Supplementary Figure S2. Scatter plots showing the Mendelian randomization analyses evaluating the association between telomere length and various hearing loss (HL) types. A, HL; B, age-related HL; C, noise-induced HL (NIHL); D, NIHL excluding heterogeneous sites rs2736176, rs3219104, rs8105767, and rs2302588. Supplementary Figure S3. “Leave-one-out” evaluation of the Mendelian randomization analyses of the associations between leukocyte telomere length and various hearing loss (HL) types. A, HL; B, age-related HL; C, noise-induced HL (NIHL); D, NIHL excluding heterogeneous sites rs2736176, rs3219104, rs8105767, and rs2302588. Supplementary Figure S4. Flow chart about the analytical methods and process of two-sample MR analysis. Supplementary Table S1. SNPs used for the LTL instruments in the Mendelian Randomization analyses. Table S2. The genetic instruments for Mendelian randomization analysis of leukocyte telomere length (exposure) and HL (outcome). Table S3. The genetic instruments for Mendelian randomization analysis of leukocyte telomere length (exposure) and ARHL (outcome). Table S4. The genetic instruments for Mendelian randomization analysis of leukocyte telomere length (exposure) and NIHL (outcome).
