# Supplementary material for: Telomere Length and Hearing Loss: A Two-Sample Mendelian Randomization
Source: Int J Environ Res Public Health. 2022 Jul 22;19(15):8937. doi: 10.3390/ijerph19158937 (PMC9330868; doi:10.3390/ijerph19158937)
Supplement: Supplementary file 1 [file ijerph-19-08937-s001.zip › Supplementary Table S1.pdf]

Table S1. SNPs used for the LTL instruments in the Mendelian Randomisation analyses.

SNPID	effect_allele	other_allele	effect_allele_freq	beta	se	P-value
rs10936600	T	A	0.243	-0.086	0.006	6.00E-51
rs7705526	A	C	0.328	0.082	0.006	5.00E-45
rs2853677	A	G	0.592	-0.064	0.005	3.00E-31
rs4691895	C	G	0.783	0.058	0.006	1.00E-21
rs9419958	C	T	0.862	-0.064	0.007	5.00E-19
rs75691080	T	C	0.091	-0.067	0.009	6.00E-14
rs59294613	A	C	0.293	-0.041	0.005	1.00E-13
rs8105767	G	A	0.289	0.039	0.005	5.00E-13
rs73624724	C	T	0.129	0.051	0.007	6.00E-12
rs3219104	C	A	0.830	0.042	0.006	9.00E-11
rs932827	T	C	0.238	-0.037	0.006	3.00E-10
rs2736176	C	G	0.313	0.034	0.005	3.00E-10
rs3785074	G	A	0.263	0.035	0.006	4.00E-10
rs7194734	T	C	0.782	-0.037	0.006	7.00E-10
rs34978822	G	C	0.015	-0.140	0.023	7.00E-10
rs34991172	G	T	0.068	-0.061	0.010	6.00E-09
rs228595	A	G	0.417	-0.028	0.005	1.00E-08
rs2302588	C	G	0.100	0.048	0.008	2.00E-08
rs13137667	C	T	0.959	0.077	0.014	2.00E-08
rs55749605	A	C	0.579	-0.037	0.007	2.00E-08
rs62053580	G	A	0.169	-0.039	0.007	4.00E-08
rs754017156	NA	GGGCCCG	0.165	0.047	0.009	8.00E-08
rs12909131	T	C	0.231	-0.031	0.006	1.00E-07
rs1744757	T	C	0.851	0.036	0.007	1.00E-07
rs2124616	A	G	0.140	-0.037	0.007	2.00E-07
rs2613954	T	C	0.886	-0.038	0.008	1.00E-06
rs12065882	G	A	0.208	0.030	0.006	1.00E-06
rs2386642	A	G	0.673	-0.026	0.005	1.00E-06
rs56810761	T	C	0.270	0.027	0.006	1.00E-06
rs62365174	G	A	0.088	-0.054	0.011	1.00E-06
rs112655343	T	C	0.102	0.043	0.009	2.00E-06
rs55710439	T	C	0.014	0.105	0.022	3.00E-06
rs11640926	G	T	0.139	0.056	0.012	3.00E-06
rs60160057	A	G	0.211	-0.029	0.006	3.00E-06
rs117536281	G	A	0.034	0.085	0.018	3.00E-06
rs7510583	G	A	0.290	0.035	0.007	3.00E-06
rs59192843	G	T	0.059	0.065	0.014	4.00E-06
rs57415150	A	G	0.042	-0.058	0.013	4.00E-06
rs6038821	T	A	0.038	0.060	0.013	4.00E-06
rs144204502	T	C	0.014	-0.090	0.020	5.00E-06
rs6107615	C	T	0.422	-0.023	0.005	6.00E-06
rs9972513	T	C	0.281	0.025	0.005	6.00E-06

rs117037102	T	C	0.018	0.098	0.022	7.00E-06
rs7276273	C	A	0.007	-0.150	0.033	7.00E-06
rs11665818	A	G	0.195	0.028	0.006	7.00E-06
rs3213718	T	C	0.583	0.022	0.005	7.00E-06
rs112347796	AG	AGAG	0.049	0.069	0.015	7.00E-06
rs143276018	C	T	0.018	-0.102	0.023	9.00E-06
rs201375979	-	TA	0.317	0.033	0.007	9.00E-06
rs7311314	A	G	0.317	0.024	0.005	9.00E-06
rs35675808	G	C	0.028	0.074	0.017	1.00E-05
rs117610974	G	C	0.009	-0.154	0.035	1.00E-05
