# Supplementary material for: Telomere Length and Hearing Loss: A Two-Sample Mendelian Randomization
Source: Int J Environ Res Public Health. 2022 Jul 22;19(15):8937. doi: 10.3390/ijerph19158937 (PMC9330868; doi:10.3390/ijerph19158937)
Supplement: Supplementary file 1 [file ijerph-19-08937-s001.zip › Supplementary Table S2.pdf]

Supplementary Table S2. The genetic instruments for Mendelian randomization analysis of leukocyte telomere length (exposure) and HL (outcome).

SNP	effect_allele	other_allele	Association with telomere length			Association with HL		
			beta	se	pval	beta	se	pval
rs2302588	C	G	0.048	0.008	2.00E-08	-0.011	0.038	0.774
rs2736176	C	G	0.034	0.005	3.00E-10	0.011	0.021	0.608
rs3219104	C	A	0.042	0.006	9.00E-11	0.068	0.094	0.470
rs4691895	C	G	0.058	0.006	1.00E-21	0.027	0.024	0.269
rs59294613	A	C	-0.041	0.005	1.00E-13	-0.009	0.023	0.675
rs62053580	G	A	-0.039	0.007	4.00E-08	0.031	0.030	0.299
rs9419958	C	T	-0.064	0.007	5.00E-19	-0.018	0.029	0.533

Notes: beta: effect size estimates; se: standard error; HL: hearing loss
