# Supplementary material for: Telomere Length and Hearing Loss: A Two-Sample Mendelian Randomization
Source: Int J Environ Res Public Health. 2022 Jul 22;19(15):8937. doi: 10.3390/ijerph19158937 (PMC9330868; doi:10.3390/ijerph19158937)
Supplement: Supplementary file 1 [file ijerph-19-08937-s001.zip › Supplementary Table S4.pdf]

Supplementary Table S4. The genetic instruments for Mendelian randomization analysis of leukocyte telomere length (exposure) and NIHL (outcome).

SNP	effect_allele	other_allele	Association with telomere length			Association with NIHL		
			beta	se	pval	beta	se	pval
rs10936600	T	A	-0.086	0.006	6.00E-51	0.000	0.001	0.690
rs13137667	C	T	0.077	0.014	2.00E-08	0.001	0.003	0.730
rs228595	A	G	-0.028	0.005	1.00E-08	-0.001	0.001	0.540
rs2302588	C	G	0.048	0.008	2.00E-08	-0.004	0.002	0.013
rs2736176	C	G	0.034	0.005	3.00E-10	0.005	0.001	0.000
rs3219104	C	A	0.042	0.006	9.00E-11	0.003	0.001	0.053
rs3219104	C	A	0.042	0.006	9.00E-11	0.003	0.005	0.530
rs3785074	G	A	0.035	0.006	4.00E-10	0.000	0.001	0.800
rs4691895	C	G	0.058	0.006	1.00E-21	0.000	0.001	0.750
rs59294613	A	C	-0.041	0.005	1.00E-13	0.000	0.001	0.790
rs62053580	G	A	-0.039	0.007	4.00E-08	0.002	0.001	0.160
rs7194734	T	C	-0.037	0.006	7.00E-10	0.000	0.001	0.920
rs75691080	T	C	-0.067	0.009	6.00E-14	0.001	0.002	0.420
rs7705526	A	C	0.082	0.006	5.00E-45	0.001	0.001	0.360
rs8105767	G	A	0.039	0.005	5.00E-13	-0.002	0.001	0.029
rs9419958	C	T	-0.064	0.007	5.00E-19	0.000	0.001	0.770

Notes: beta: effect size estimates; se: standard error; NIHL: noise-induced hearing loss
