# Supplementary figures and images for: Telomere Length and Hearing Loss: A Two-Sample Mendelian Randomization
Source: Int J Environ Res Public Health. 2022 Jul 22;19(15):8937. doi: 10.3390/ijerph19158937 (PMC9330868; doi:10.3390/ijerph19158937)

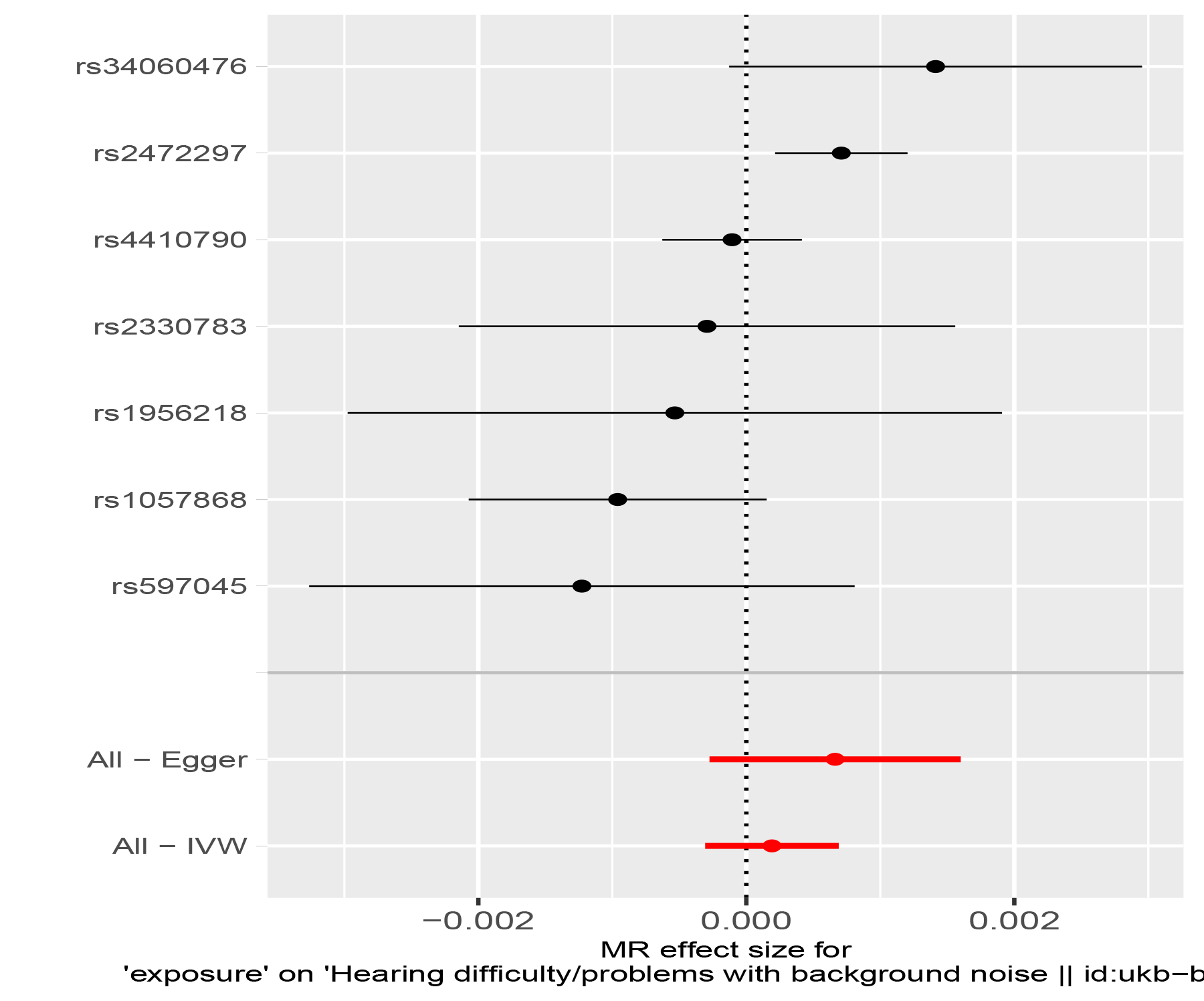

Supplement: Supplementary file 1 [file ijerph-19-08937-s001.zip › Supplementary_Figures/Supplementary figures S1(A).tif]

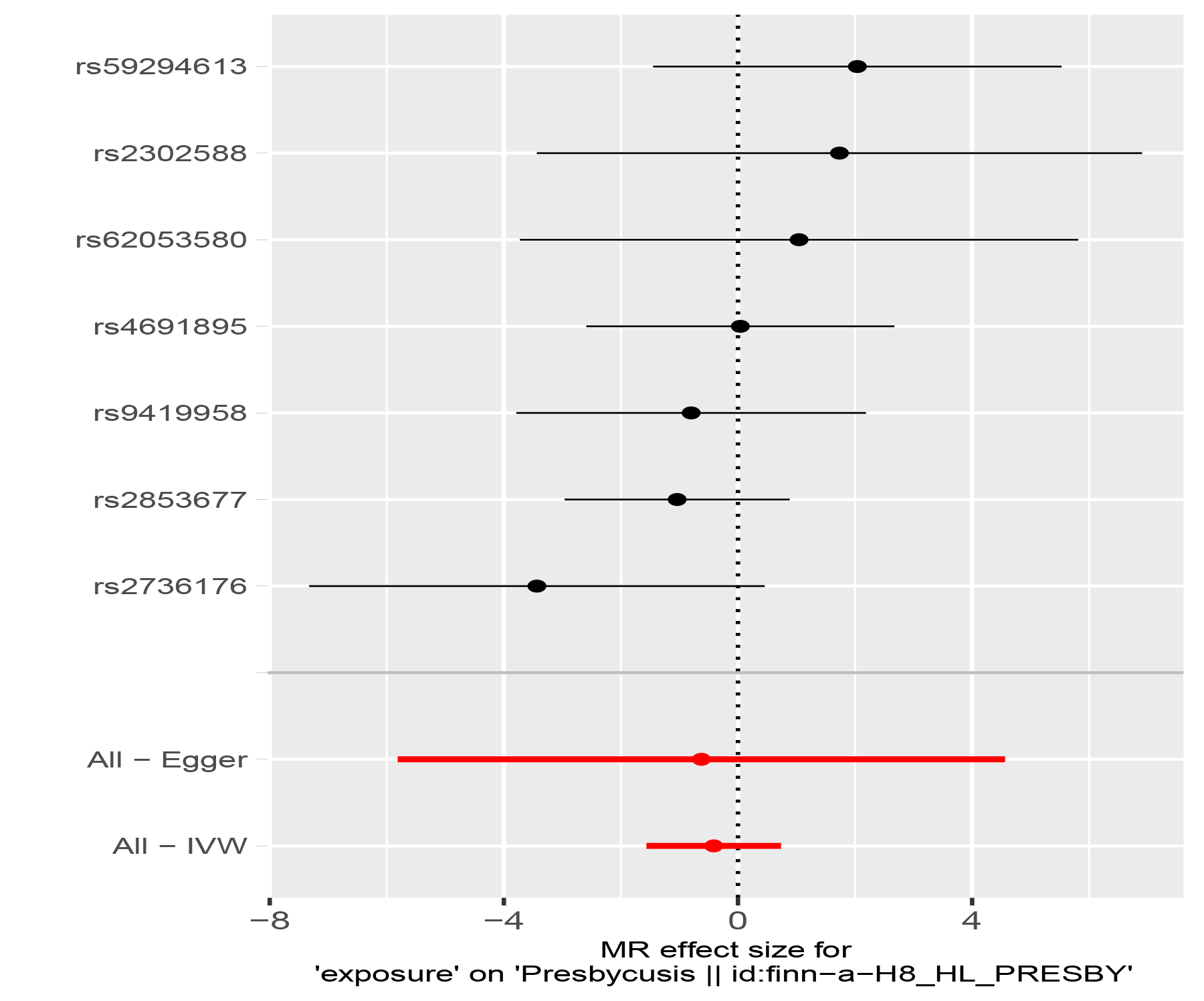

Supplement: Supplementary file 1 [file ijerph-19-08937-s001.zip › Supplementary_Figures/Supplementary figures S1(B).tif]

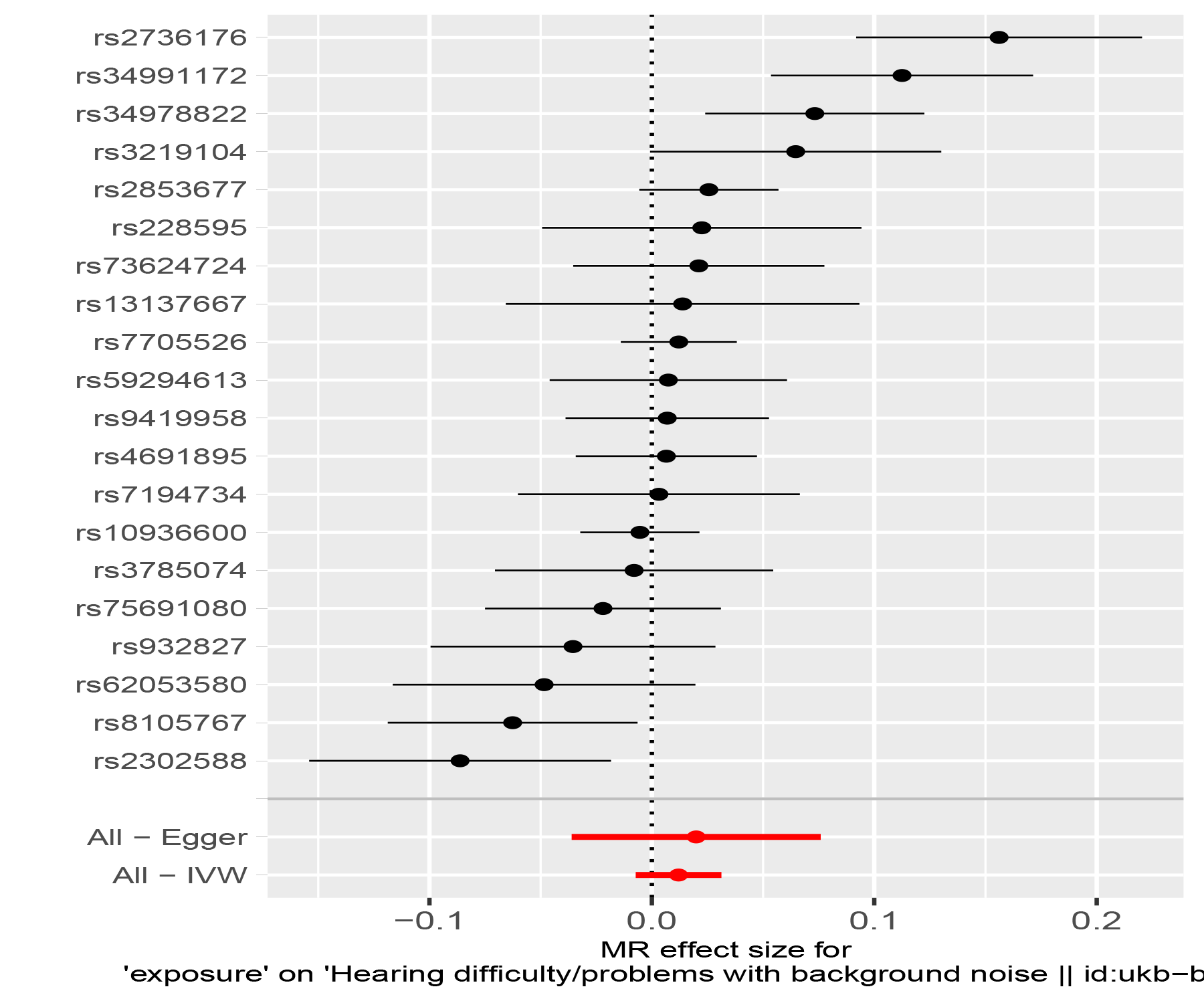

Supplement: Supplementary file 1 [file ijerph-19-08937-s001.zip › Supplementary_Figures/Supplementary figures S1(C).tif]

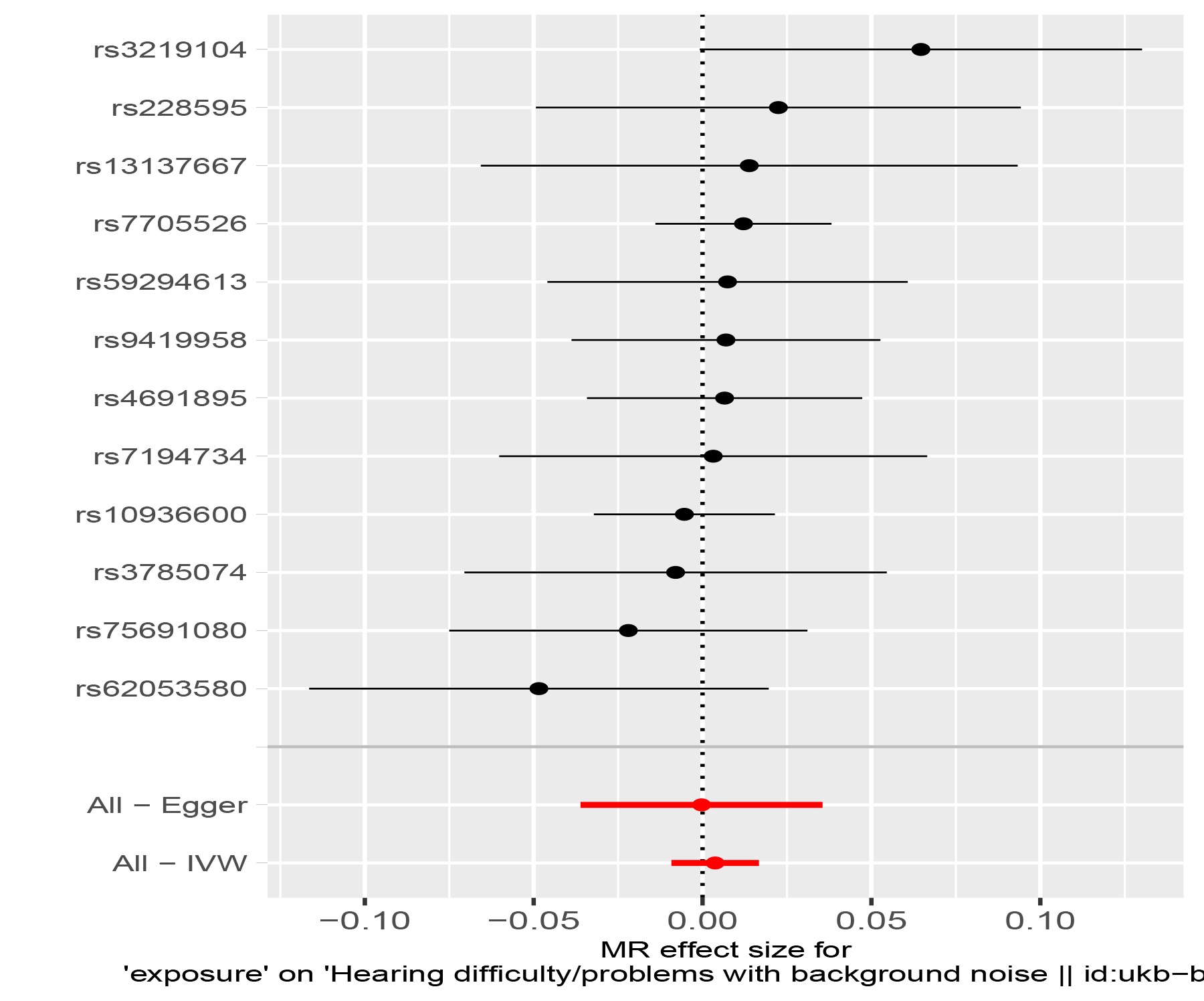

Supplement: Supplementary file 1 [file ijerph-19-08937-s001.zip › Supplementary_Figures/Supplementary figures S1(D).tif]

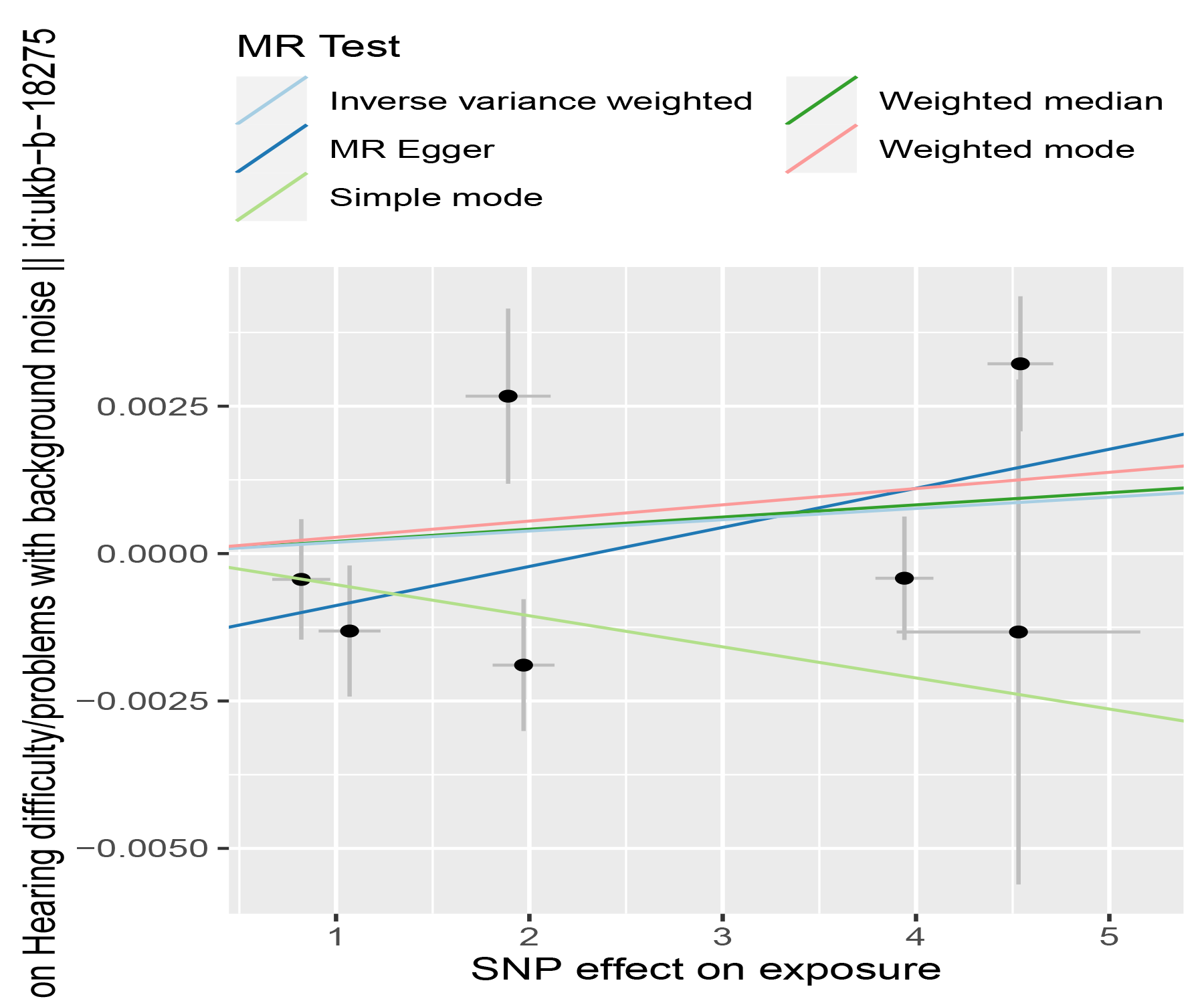

Supplement: Supplementary file 1 [file ijerph-19-08937-s001.zip › Supplementary_Figures/Supplementary figures S2(A).tif]

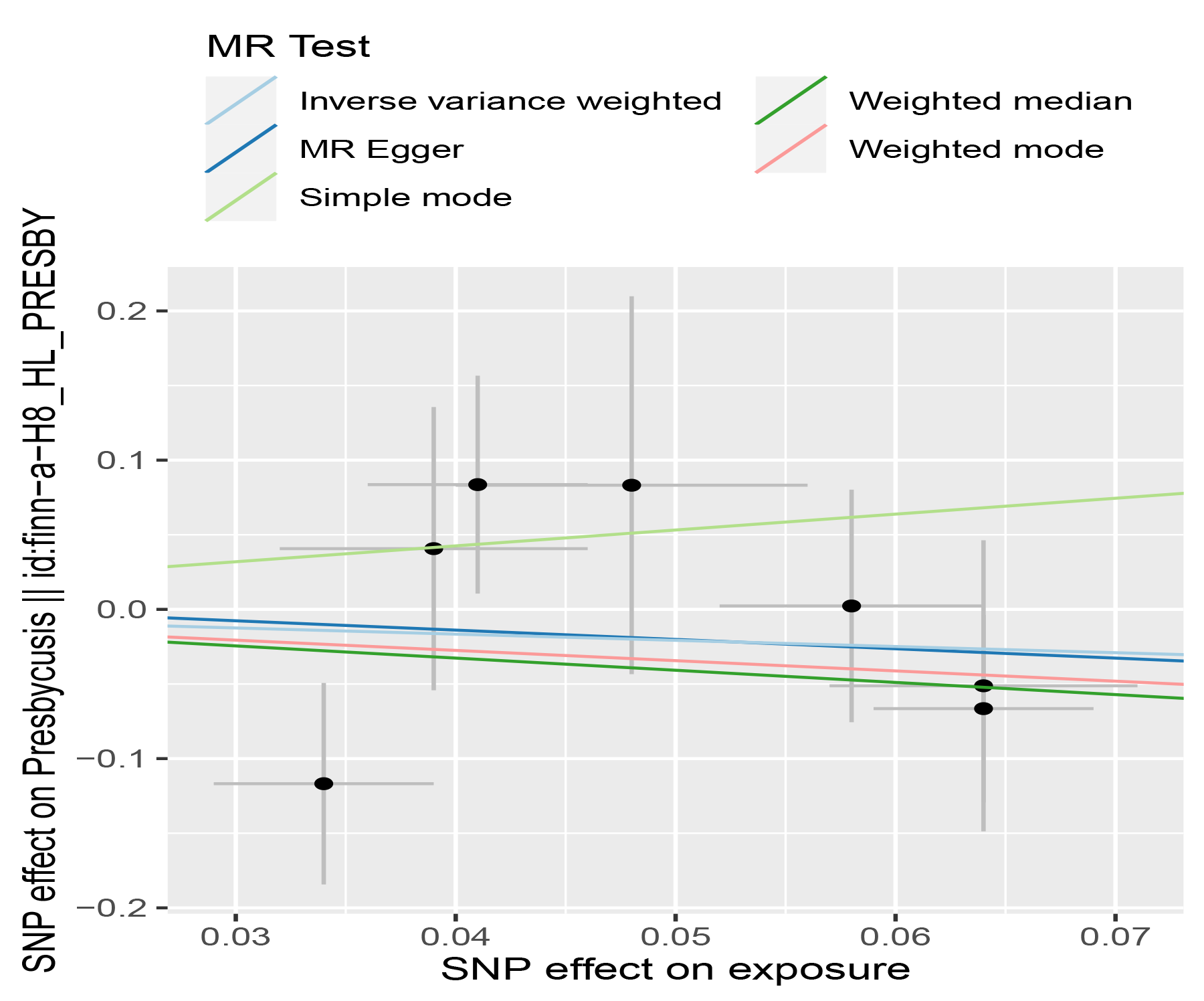

Supplement: Supplementary file 1 [file ijerph-19-08937-s001.zip › Supplementary_Figures/Supplementary figures S2(B).tif]

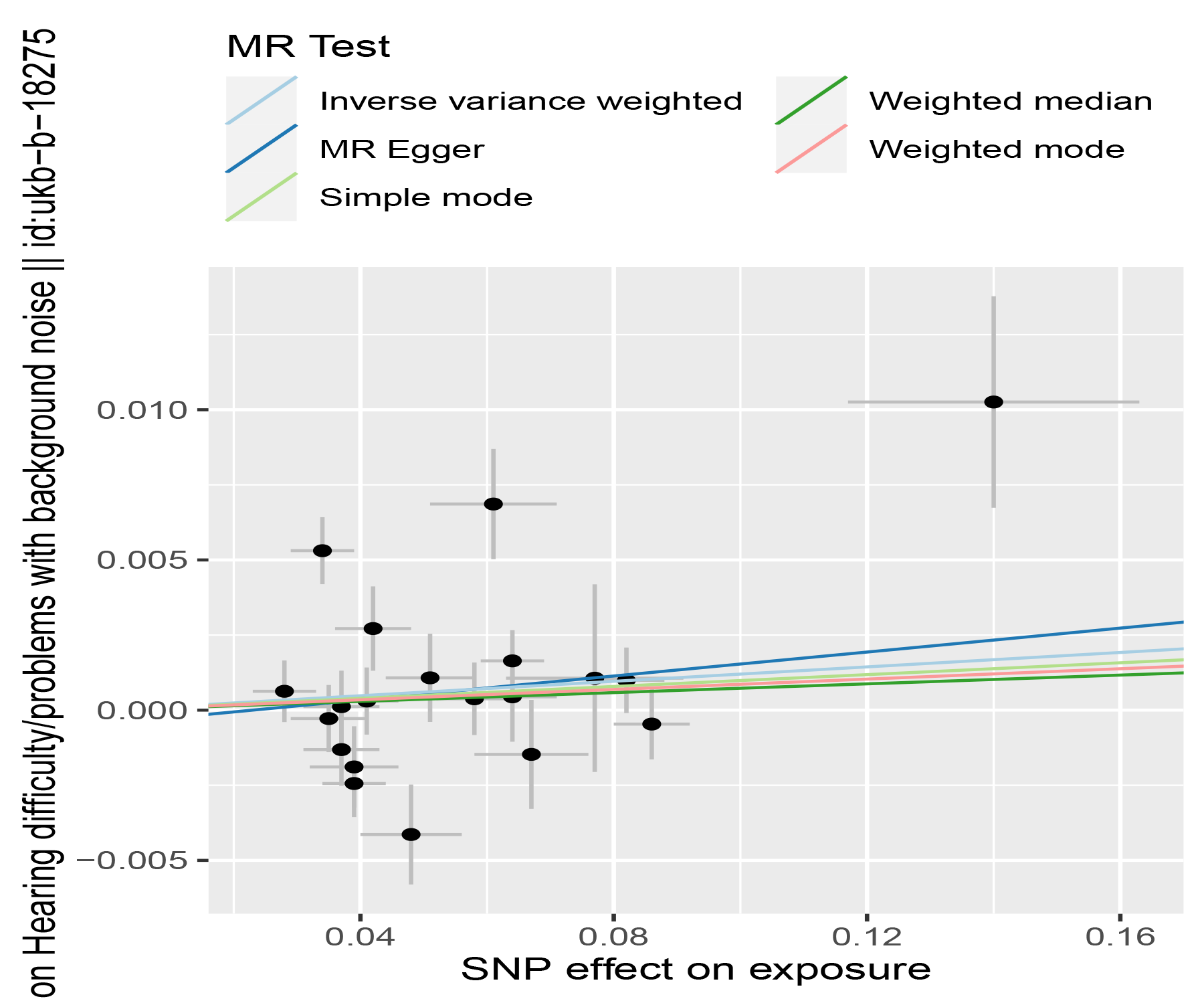

Supplement: Supplementary file 1 [file ijerph-19-08937-s001.zip › Supplementary_Figures/Supplementary figures S2(C).tif]

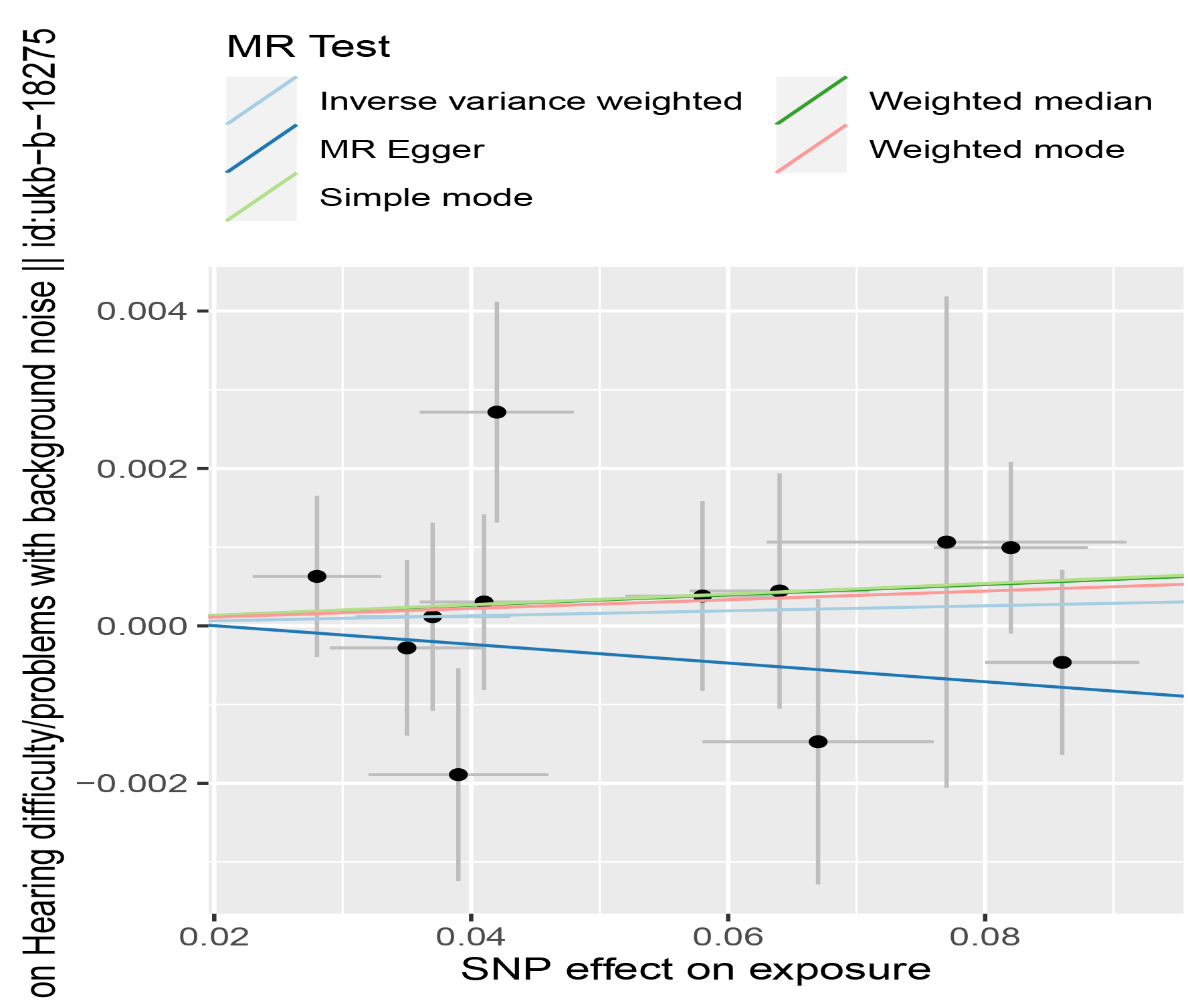

Supplement: Supplementary file 1 [file ijerph-19-08937-s001.zip › Supplementary_Figures/Supplementary figures S2(D).tif]

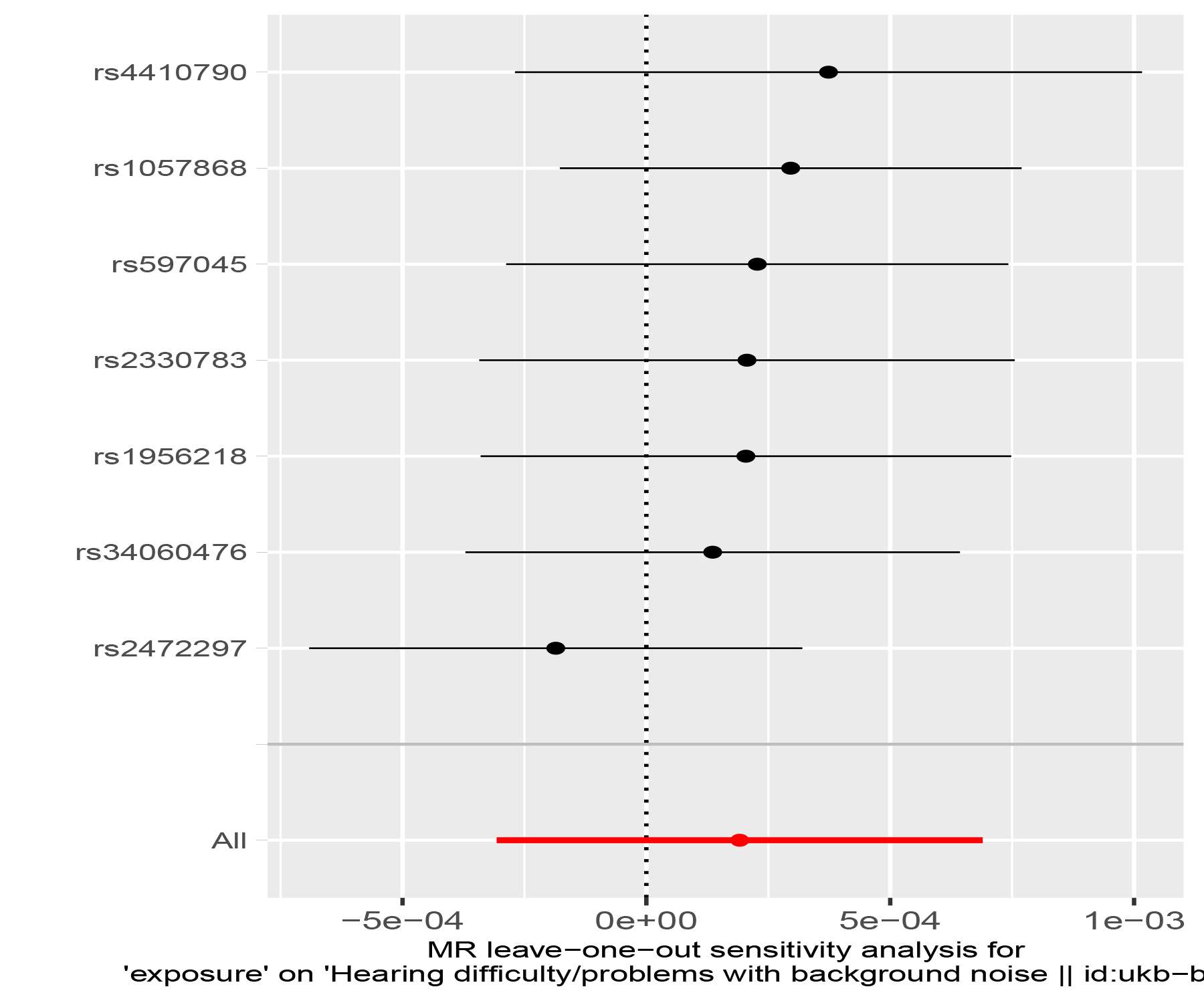

Supplement: Supplementary file 1 [file ijerph-19-08937-s001.zip › Supplementary_Figures/Supplementary figures S3(A).tif]

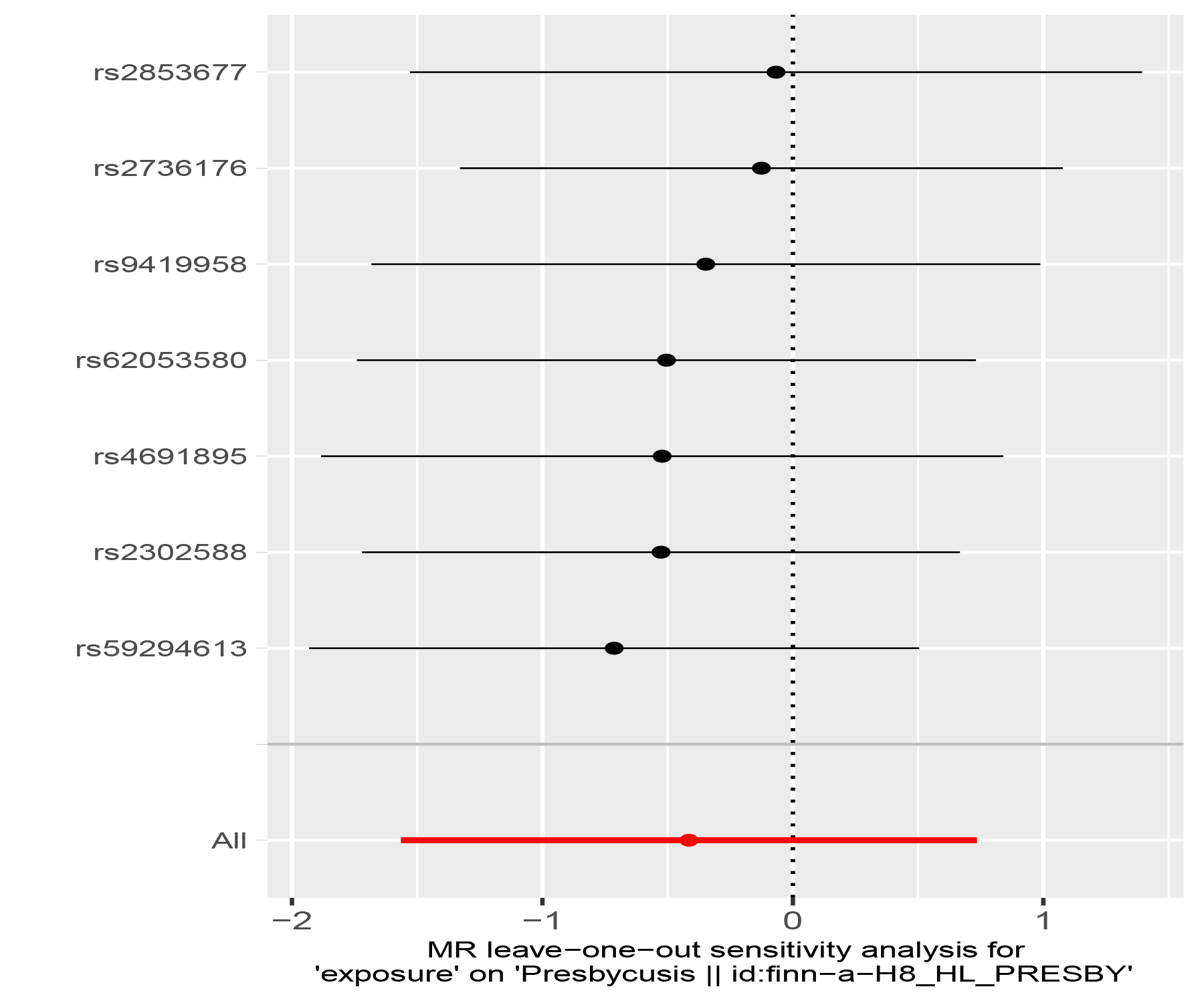

Supplement: Supplementary file 1 [file ijerph-19-08937-s001.zip › Supplementary_Figures/Supplementary figures S3(B).tif]

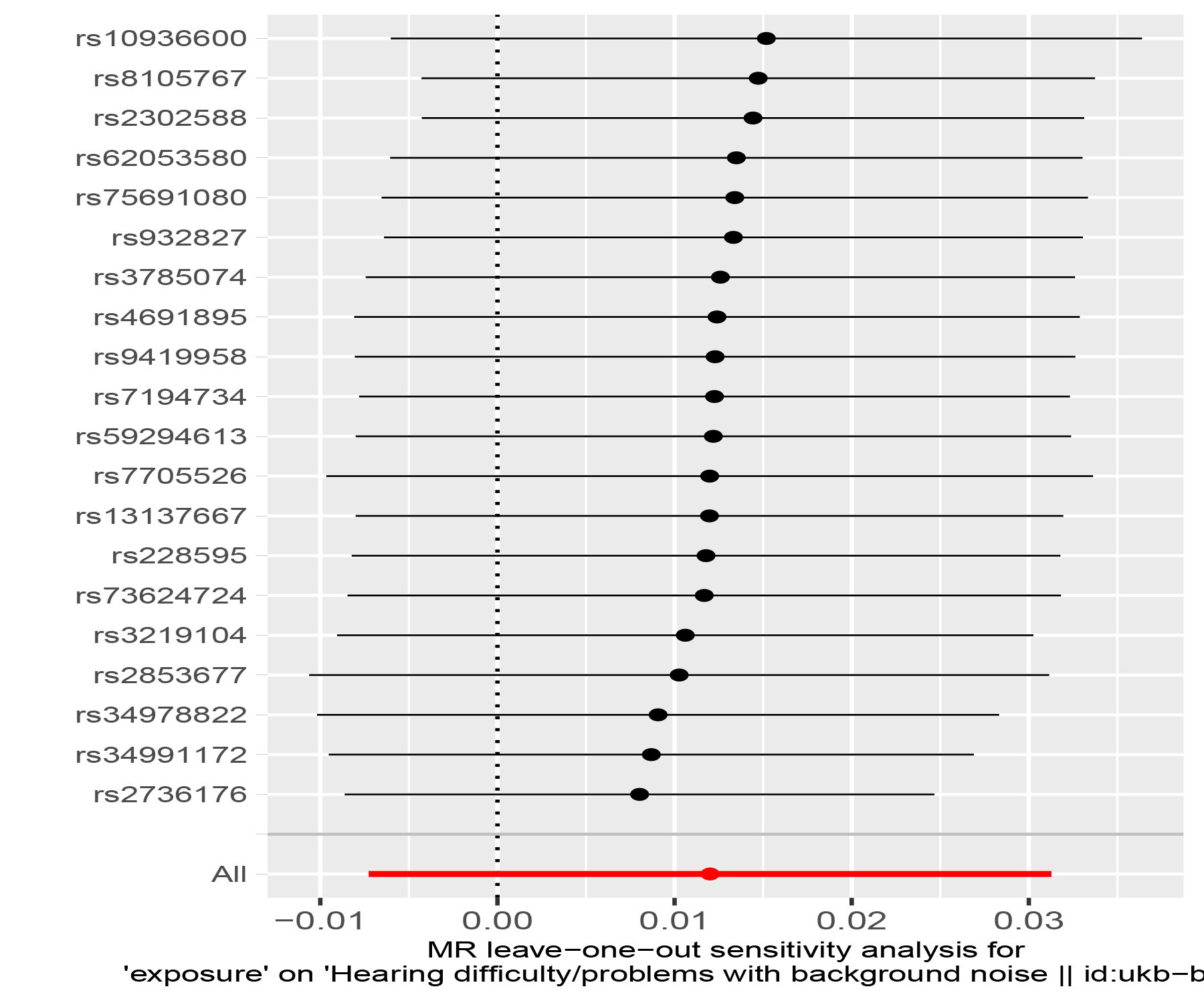

Supplement: Supplementary file 1 [file ijerph-19-08937-s001.zip › Supplementary_Figures/Supplementary figures S3(C).tif]

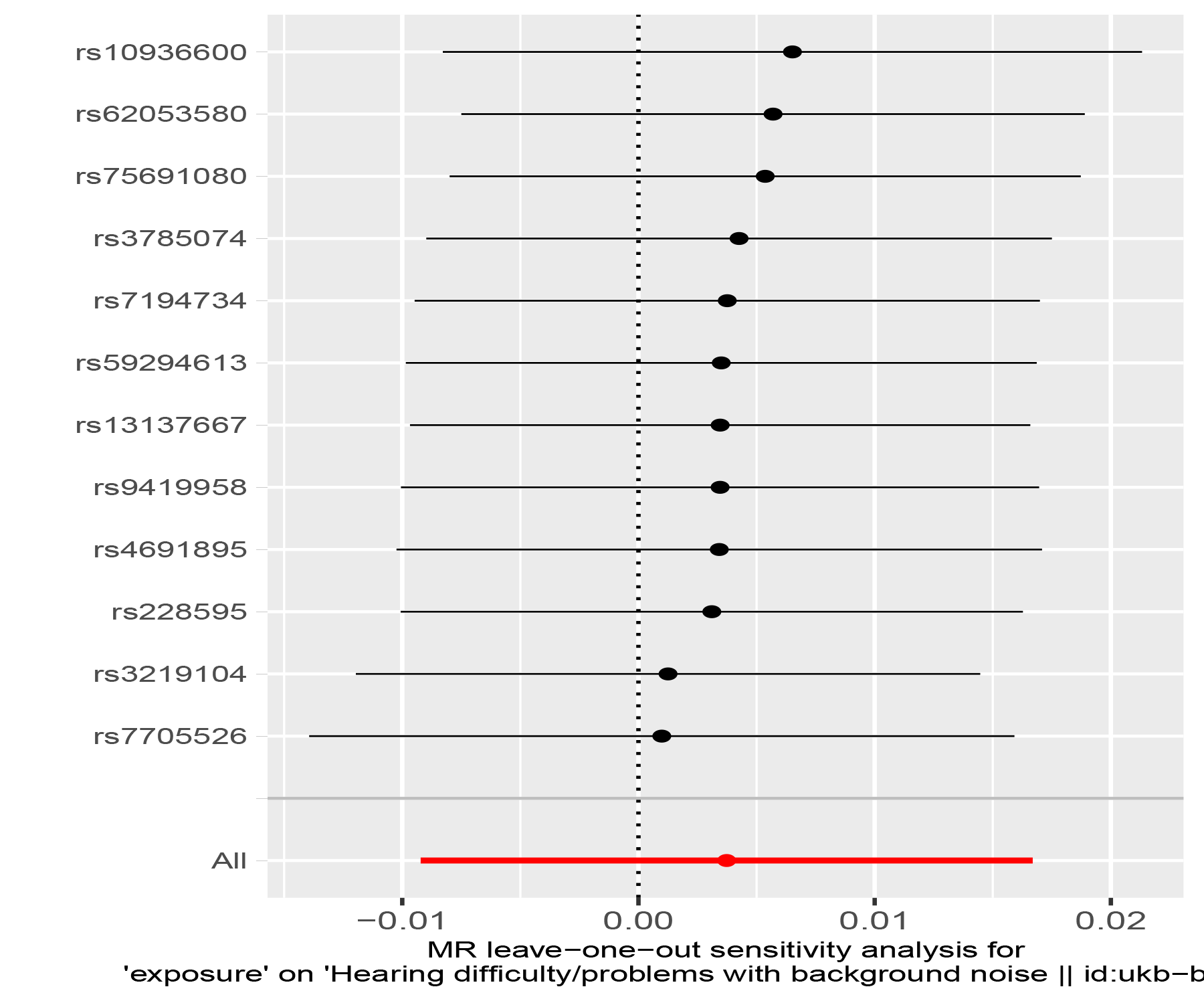

Supplement: Supplementary file 1 [file ijerph-19-08937-s001.zip › Supplementary_Figures/Supplementary figures S3(D).tif]

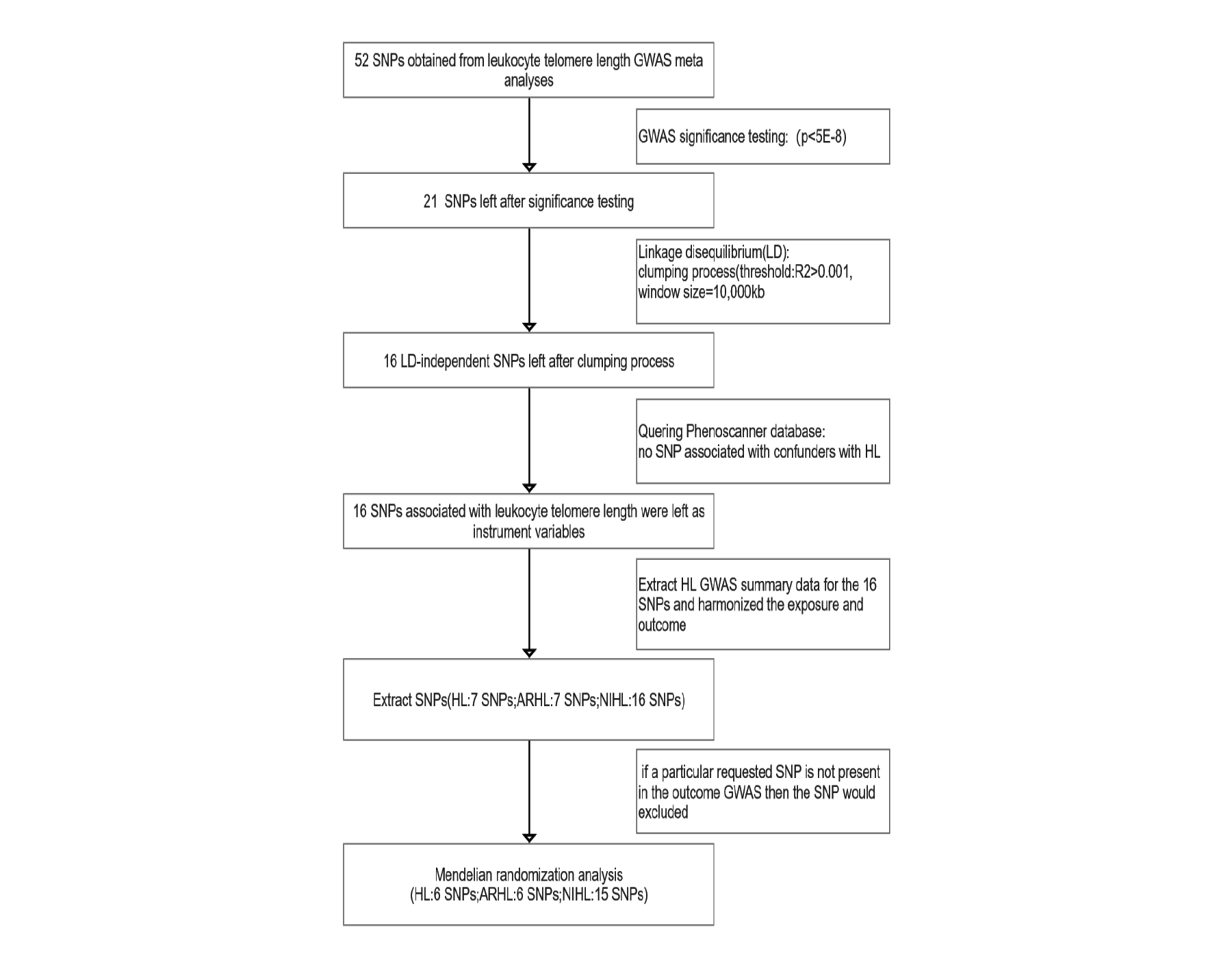

Supplement: Supplementary file 1 [file ijerph-19-08937-s001.zip › Supplementary_Figures/Supplementary figures S4.tif]
